# Prevalence and Risk Factors of Cytopenia in HIV-Infected Patients before and after the Initiation of HAART

**DOI:** 10.1155/2020/3132589

**Published:** 2020-01-28

**Authors:** Lina Fan, Cuilin Li, Hongxin Zhao

**Affiliations:** Clinical and Research Center of Infectious Diseases, Beijing Ditan Hospital, Capital Medical University, Beijing 100015, China

## Abstract

**Background:**

Cytopenia is a frequent hematological disorder in patients with human immunodeficiency virus (HIV) infection. However, the distribution and risk factors of cytopenia in patients starting highly active anti-retrovirus treatment (HAART) and the effect of HAART on cytopenia were not fully investigated.

**Methods:**

From November 2004 to August 2016, a retrospective study was conducted to evaluate the prevalence of cytopenia in 4325 HAART-naïve patients. Risk factors of cytopenia at baseline and on recovery from cytopenia were analyzed using logistic regression analysis after 24 months of HAART in Beijing Ditan Hospital.

**Results:**

The prevalence of cytopenia was 19.1% in HIV-naïve patients. Risk factors for cytopenia in HAART-naïve patients were a CD4 cell count<200 cells/*μ*L, femaleness, WHO stage IV, coinfection with hepatitis B virus (HBV), BMI <18.5 kg/m^2^, a viral load ≥100,000 copies/ml, and age ≥40 years. In total, 70.2% and 76.4% of patients with cytopenia recovered after 6 and 24 months of HAART, respectively. The predictors of patients without normal blood cells after 24 months HAART were a CD4 cell count of <200 cells/*μ*L, femaleness, WHO stage IV, coinfection with hepatitis B virus (HBV), BMI <18.5 kg/m^2^, a viral load ≥100,000 copies/ml, and age ≥40 years. In total, 70.2% and 76.4% of patients with cytopenia recovered after 6 and 24 months of HAART, respectively. The predictors of patients without normal blood cells after 24 months HAART were a CD4 cell count of <200 cells/

**Conclusion:**

Early detection could decrease the prevalence of HIV-related cytopenia, while starting HAART as early as possible seems to be effective for normalization of the blood cells in HIV-infected patients.

## 1. Introduction

Cytopenia is a frequent hematological disorder in patients with human immunodeficiency virus (HIV) infection. The most common manifestation is reduction of any of the blood cell lines, leading to neutropenia, anemia, and thrombocytopenia. Previous studies have reported prevalence rates of anemia, neutropenia, and thrombocytopenia of 1.3%–95% [1], 10%–85% [2, 3], and 7%–21% [4, 5], respectively; the wide span reflects the different definitions, geographical locations, race/ethnicity of patients, and stages of disease. Lower CD4^+^ T-cell counts, higher viral loads, advanced disease stages, and side effects of medicines used for HIV were risk factors for cytopenias in HIV-infected patients [5].

In HIV-infected patients, cytopenia has been associated with the progression to AIDS (anemia), high mortality (anemia), hospitalization (all), and secondary infections caused by bacterial and fungal pathogens (neutropenia) or drugs used for HIV [6–11]. Highly active antiretroviral therapy (HAART) has been proven to reduce AIDS-related mortality significantly and leads to improvements in cytopenia [9, 12–16]. However, some antiretroviral compounds such as zidovudine (AZT) also exert bone marrow cytotoxicity and contribute to cytopenia in HIV-infected patients [9, 13, 17]. Monitoring changes in hematological parameters in HIV-infected patients while they are on HAART could be a useful indicator of the patient response to HAART.

In China, Dai et al. found that the prevalence of anemia was 9.76% [6], while Fan et al. reported a thrombocytopenia rate of 4.5% among 1730 patients [18]. Shen reported that the prevalence rates of leukopenia and thrombocytopenia were 33.2% and 15.6% [19]. However, the prevalence rates and risk factors of cytopenia and the effect of HAART on cytopenia have not been well documented. This study was designed to investigate the prevalence and risk factors of cytopenia among HAART-naïve patients. Furthermore, we aimed to evaluate the effect of HAART on cytopenia.

## 2. Materials and Methods

### 2.1. Study Subjects

In total, 5047 HIV-infected patients who were on follow-up after HAART at the Department of Infectious Diseases, Beijing Ditan Hospital, Capital Medical University, from November, 2004 to August, 2016, were included in this study. This study was approved by the ethics committees of Beijing Ditan Hospital, Capital Medical University, and the study conformed to the Declaration of Helsinki. The profiles of 722 participants were excluded because of neutrophilia (*n* = 108) or incomplete information (*n* = 613). Finally, a total of 4325 patients were included. The demographic and clinical data for the HAART-naïve patients including sex, age, height, weight, route of transmission, the initial time of HAART treatment, and WHO stage were collected. Laboratory data such as absolute neutrophil numbers, hemoglobin concentration, absolute platelet count, and absolute CD4^+^ T-cell count were collected at baseline.

**Figure 1:**
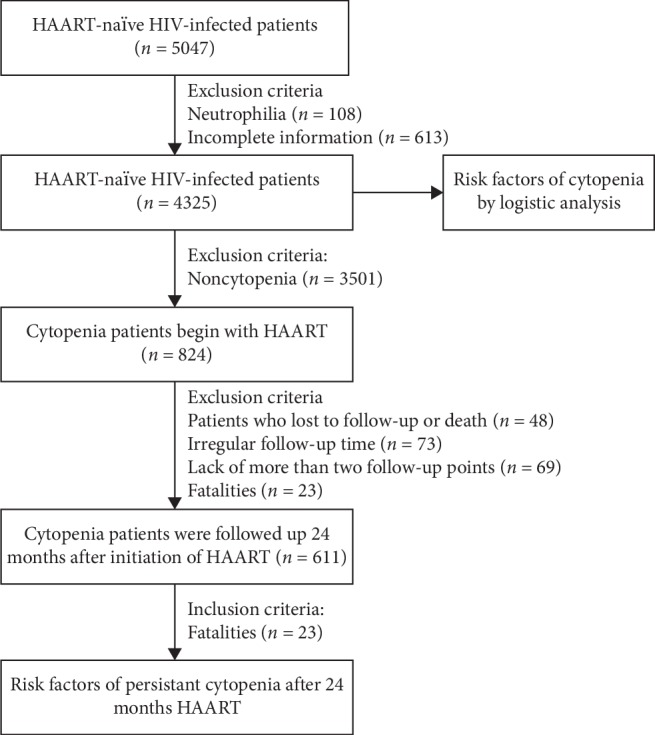
Flow chart of this study.

To investigate the effect of HAART on cytopenia, 824 HIV-infected patients with cytopenia were followed up to 24 months after the initiation of HAART. The exclusion criteria were all patients without cytopenia *n* = 3501, patients who were lost at follow-up *n* = 48, fatalities *n* = 23, irregular follow-up times *n* = 73, and lack of follow-up time points (>2 follow-up time points) *n* = 69. In total, 213 patients were excluded and 611 HIV-infected patients with cytopenia were included. The number of neutrophils, the platelet count, the CD4^+^ T-cell count, and the hemoglobin concentration at 6, 12 and 24 months after the initiation of HAART were recorded. The flow chart is shown in Figure 1.

### 2.2. Definition

Conditions were defined based on the following parameters. Anemia: hemoglobin <11 g/dL in women or <12 g/dL in men [20]; neutropenia: neutrophil count <2000 cells/*μ*L; thrombocytopenia: platelet count <100,000 cells/*μ*L. Patients with isolated anemia, thrombocytopenia or neutropenia were defined as patients with unicytopenia. Bicytopenia was defined as a patient in whom any two of the three lineage cell counts (neutrophils, hemoglobin, or platelets) were below the levels designated above. Pancytopenia was defined as having three lineage cell counts (neutrophils, hemoglobin, or platelets) below the levels designated above.

### 2.3. Data Collection

Retrospective data were collected from medical records, including demographic, clinical, and laboratory characteristics. Variables include age, sex, HIV transmission route, coinfection with HBV, coinfection with HCV, WHO staging, HIV viral load, the use of AZT and stavudine (D4T), and body mass index (BMI). Age was categorized as <40 and over 40 years. Based on Chinese guidelines for diagnosis and treatment of HIV/AIDS, CD4 T-cell count was categorized as <200 cells/*μ*l and over 200 cells/*μ*l [21]. Based on the WHO clinical staging of HIV disease in adults and adolescents, subjects were classified in to stage I, stage II, stage III, and stage IV [22]. The cutoff for BMI for defining underweight was <18.5 kg/m^2^ and overweight and obesity was >24.0 kg/m^2^ [23, 24]. HIV viral load was categorized as <100000 copies/ml and over 100000 copies/ml [25].

### 2.4. Statistical Analysis

All statistical analyses were performed using SPSS 22.0 software (SPSS, Chicago, IL, USA) and GraphPad 7 (GraphPad Software, La Jolla, CA, USA). Data were presented as the medians with interquartile ranges (IQR) due to skewed statistical distributions, while categorical variables were presented by percentages. The differences between two groups were analyzed using the Mann–Whitney *U* test (nonparametric). A Pearson's test was used to evaluate differences in categorical variables. Binary logistic regression analysis or ordinal logistic regression analysis was used to evaluate risk factors associated with cytopenias and persistent cytopenia after 24 months of HAART. Demographic variables (sex and age) and clinical/laboratory characteristics (CD4^+^ T-cell count, BMI, WHO staging, viral load, transmission routes, coinfection with HBV, and coinfection with HCV) were included to investigate for the risk factor of cytopenia. The risk factors of persistent cytopenia investigated included demographic variables (sex and age) and clinical/laboratory variables (CD4^+^ T-cell count at baseline and after 24 months HAART, BMI, WHO staging at baseline, coinfection with HBV, and coinfection with HCV, viral load, and the use of AZT and D4T). Statistical significance was set at a level of *p* < 0.05.

## 3. Results

### 3.1. The Demographic and Clinical Characteristics of HAART-Naïve Patients

A total of 4325 individuals were included in this study, of which 26.1% had a CD4 cell count of <200 cells/*μ*L. The median age was 36 years, the ratio of males to females was 15 : 1, the median BMI was 21.5 (19.7, 23.7) kg/m^2^, and the median CD4^+^ T-cell count was 249 (151, 356) cells/*μ*L. Sexual transmission was mainly via the transmission route, which accounted for 91% of cases. According to WHO staging, stages II and III were present in 2541 (81.9%) individuals (Table 1).

### 3.2. The Prevalence and Distribution of Cytopenia in HAART-Naïve Patients

**Figure 2:**
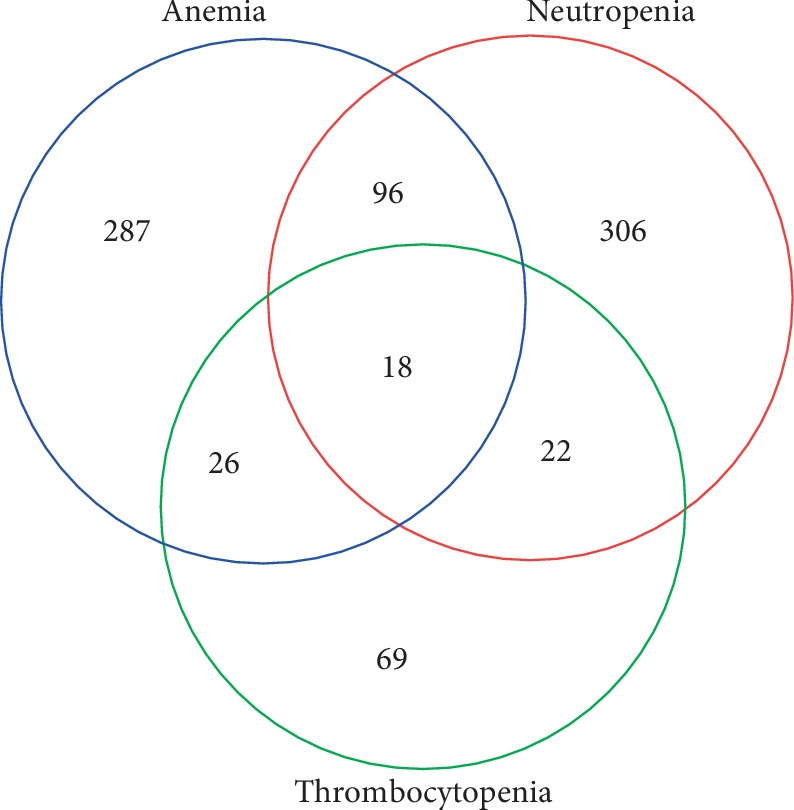
Distribution of cytopenia cases among the participants of the study.

Cytopenia was detected in 824 (19.1%) patients in our cohort, of which 662 (15.3%) patients had unicytopenia, 144 (3.3%) had bicytopenia, and 18 (0.4%) had pancytopenia. Among the unicytopenia cases, 287 (6.63%) patients had isolated anemia, 306 (7.7%) had isolated neutropenia, and 69 (1.6%) had isolated thrombocytopenia. Among the bicytopenia cases, 96 (2.2%) had anemia and neutropenia, 26 (0.6%) had anemia and thrombocytopenia, and 22 (0.5%) had thrombocytopenia and neutropenia. The prevalence and distribution of cytopenia are shown in Figure 2.

### 3.3. The Risk Factors for Cytopenia in HAART-Naïve Patients

**Figure 3:**
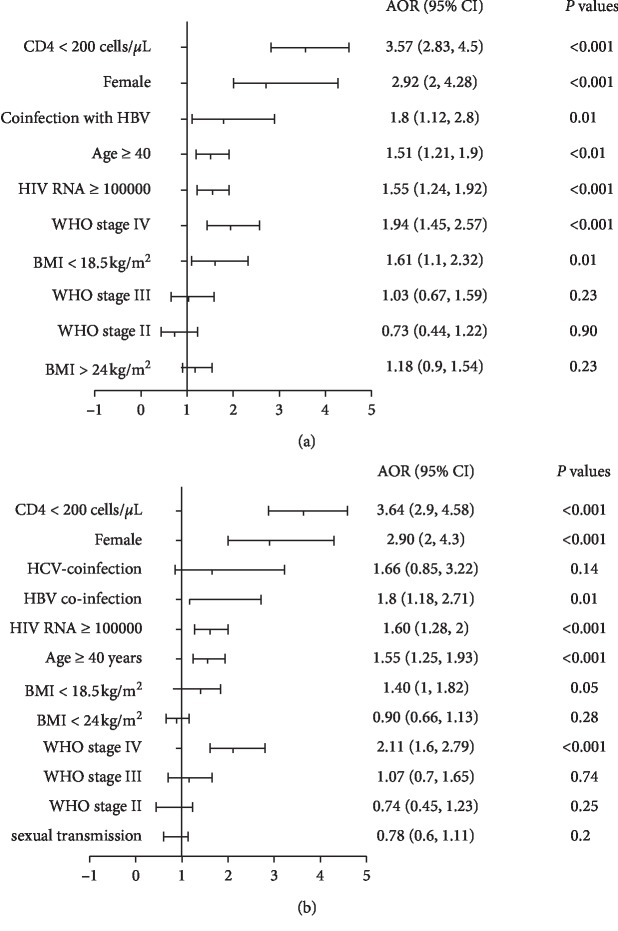
The forest plot of logistic analysis for cytopenias at baseline. (a) Binary logistic regression analysis for cytopenia. (b) Ordinal logistics regression analysis for unicytopenia, bicytopenia, and pancytopenia.

As seen in Figure 3, multivariate logistic regression analyses were performed to investigate the risk factors of cytopenia in HAART-naïve patients. We found that a CD4 cell count <200 cells/*μ*L (OR: 3.57, 95% CI: 2.83–4.5, *p* < 0.001), femaleness (OR: 2.92, 95% CI: 2–4.28, *p* < 0.001), WHO stage IV (OR: 1.94, 95% CI: 1.45–2.57, *p* < 0.001), coinfection with hepatitis B virus (HBV) (OR: 1.8, 95% CI: 1.12–2.9, *p*=0.007), BMI <18.5 kg/m^2^ (OR 1.61, 95% CI: 1.1–2.32, *p*=0.01), a viral load >100,000 (OR: 1.55, 95% CI: 1.24–1.92, *p* < 0.001), and age ≥40 years (OR: 1.51, 95% CI: 1.21–1.9, *p* < 0.01) were all risk factors of cytopenia (Figure 3(a); Table 2). Cytopenia was not associated with HCV coinfection or transmission route.

Considering the distribution of cytopenia, we chose the ordinal logistic regression model to test the risk factors of blood cell line reduction. Our results showed that lower CD4 cell count <200 cells/*μ*L (OR: 3.64, 95% CI: 2.9–4.58, *p* < 0.001), femaleness (OR: 2.9, 95% CI: 2–4.3, *p* < 0.001), WHO stage IV (OR: 2.11, 95% CI: 1.6–2.79, *p* < 0.001), coinfection with hepatitis B virus (HBV) (OR: 1.8, 95% CI: 1.18–2.71, *p*=0.01), BMI <18.5 kg/m^2^ (OR 1.4, 95% CI: 1.1–1.82, *p*=0.048), a viral load >100,000 (OR: 1.6, 95% CI: 1.28–2, *p* < 0.001), and age ≥40 years (OR: 1.55, 95% CI: 1.25–1.93, *p* < 0.001) were all risk factors of cytopenias (Figure 3(b); Table 3).

### 3.4. Effect of HAART on Cytopenia

**Figure 4:**
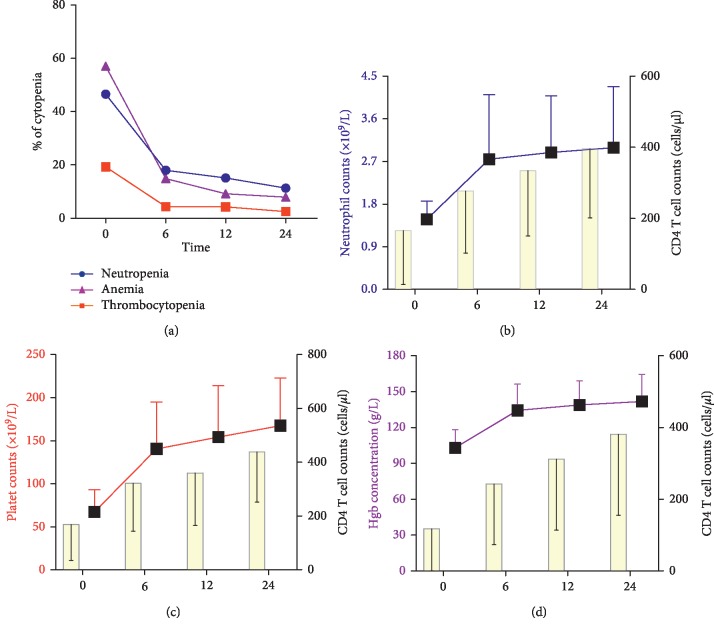
Recovery from cytopenia in patients before the initiation of HAART and after 6, 12, and 24 months of HAART. (a) Recovery from neutropenia, thrombocytopenia, and anemia in patients. (b) Absolute neutrophil count and absolute CD4^+^ T-cell count in patients with neutropenia. (c) Absolute platelet counts and absolute CD4^+^ T-cell count in patients with thrombocytopenia. (d) Hemoglobin concentration and the absolute count of CD4^+^ T cells in patients with anemia.

Obvious resolution of cytopenia was observed after 24 months of HAART. The percentage of cytopenia recovery is shown in Table 4. We found that the proportions of neutropenia, anemia, thrombocytopenia, bicytopenia, and pancytopenia were all decreased after 6, 12, and 24 months of HAART, as shown in Figure 4(a). After antiviral treatment, with the increase in the CD4^+^ T-cell count, the absolute count of neutrophils, the absolute platelet counts, and the hemoglobin concentration in cytopenia patients increased sharply up to 6 months of HAART and then slowly after 6 months of therapy (Figures 4(b)–4(d)).

### 3.5. The Risk Factors for Persistent Cytopenia after 24 Months HAART

To investigate the risk factors for these patients without persistent cytopenia, a logistic regression analysis was performed. We found that a CD4 cell count of <200 cells/*μ*L at baseline (OR: 2.42, 95% CI: 1.2–4.86, *p*=0.013), femaleness (OR: 5.54, 95% CI: 2.47–12.42, *p* < 0.001), coinfection with HBV (OR: 4.84, 95% CI: 1.47–15.91, *p*=0.01), and treatment with AZT (OR: 3.71, 95% CI: 1.16–11.9, *p*=0.009) were all risk factors for unrecoverable hematological parameters after 24 months of antiviral therapy (Table 5).

## 4. Discussion

In the present study, we found that HAART was an effective treatment for cytopenia patients before the initiation of HAART. Along with the recovery of the CD4^+^ T-cell count, blood cells (neutrophils, platelets, and hemoglobin) were also increased. Femaleness, the lower CD4^+^ T-cell count at baseline, an advance stage at baseline, and coinfection with HBV were risk factors for persistent cytopenia after HAART, which suggested the importance of starting HAART early in cytopenia patients with HIV infection.

There have been a large number of studies about the prevalence of anemia, thrombocytopenia, leucopenia, and neutropenia [26–30], and HIV-1-infected patients often show a reduction in two or three blood cell lines. Furthermore, pancytopenia was linked with higher mortality than nonpancytopenia [31]; however, there have been few studies to investigate this fully. In this study, we found that the most common type of cytopenia was unicytopenia, followed by bicytopenia and pancytopenia. Among unicytopenia, the neutropenia was the most common cytopenia and neutropenia and anemia was the most common bicytopenia.

In this study, CD4 <200 cells/*μ*l, advanced WHO clinical stage, higher HIV viral load, age ≥40 years, and coinfection with HBV at baseline was identified as the risk factors for cytopenia in the multivariate analysis, a finding consistent with that of the previous studies [32]. The association of low CD4 cell count with cytopenia may be due to the effect of HIV on the function of early hematopoietic progenitor cells [33].

Previous studies have reported that HAART is an effective treatment for anemia, thrombocytopenia, and neutropenia [16, 28, 34] in HIV-1-infected patients, and the results of our study were consistent with this. We found that the neutrophil count, the platelet count, and the hemoglobin concentration were increased and the proportions of neutropenia, anemia, and thrombocytopenia were decreased after 6 months of HAART. The resolution of cytopenia was associated with the improvement in the CD4^+^ T-cell count, which indicated that HIV-related cytopenia was caused by HIV-1 and immunosuppression and that virus suppression and immunological reconstitution could prompt the normalization of blood cells.

In accordance with a previous study, we also found that a low CD4^+^ T-cell count and advanced staging before the initiation of HAART were predictors of patients with persistent cytopenia after 24 months of HAART. Levine et al. found that HAART use and higher CD4^+^ T-cell counts were associated with the resolution of neutropenia [34]. Berhane et al. reported that a CD4 cell count <200 cells/*μ*L, HIV RNA >5000 copies/mL, and erythrocyte mean corpuscular volume <80 fl were independent predictors of anemia after 12 months of HAART [14]. O'Bryan et al. demonstrated that patients with a higher HIV load were at risk of persistent thrombocytopenia after HAART [15]. In the present study, we also found that a low CD4^+^ T-cell count was associated with HAART-naïve patients with cytopenias, which suggested the advanced stage before initiation of HAART was associated with the risk of cytopenia and the recovery ratio of cytopenia after treatment. More importantly, early detection of HIV could decrease the prevalence of cytopenias, while early initiation of HAART was very important for HIV-1 infected patients with cytopenia.

A previous study found that the incidence of cytopenia was 63.2% in HIV-infected patients in South Africa [35]. Kyeyune et al. reported that the prevalence of cytopenia was 65%, of which 21.9% of patients had bicytopenia and only 2/400 patients had pancytopenia [5]. In the present study, we found that the prevalence of cytopenia was 19.1%, and the difference between this result and that of the former study might be due to differences in the population and the geographical location.

The present study found that the ratio of recovery was 68.2% in patients with cytopenia after 6 months HAART, and 76.4% of patients with cytopenia returned to normal blood cell levels after 24 months HAART. After 6 months of antiviral therapy, the increase in blood cells obviously slowed down. The main reasons for this were that viral replication was suppressed and immunity was improved by 6 months of HAART, and HIV-1 related cytopenia caused by myelosuppression was relieved. Other factors such as malnutrition and a patient's economic situation may play a role in persistent cytopenia after 6 months of HAART.

It is not only HIV-1, but also HAART such as AZT, that can lead to persistent hematopoietic suppression and resulting cytopenia [3, 17]. In our study, among cytopenia patients with persistent cytopenia after 24 months of HAART, AZT was used in 28 patients. We also found that persistent cytopenia was associated with the effects of AZT.

There are some limitations to our study. First, there was potential inherent bias in a retrospective observational study. Second, most of the participants in the present study were urban residents of Beijing, who are not necessarily representative of all HIV-infected patients in China.

## 5. Conclusion

The prevalence of cytopenia in patients before the initiation of HAART was 19.1%, of which the prevalence of unicytopenia, bicytopenia, and pancytopenia was 15.3%, 3.3%, and 0.4%, respectively. Cytopenia was associated with femaleness, a lower BMI, a lower CD4^+^ T-cell count, a higher viral load, WHO stage IV, and coinfection with HBV. HAART was an effective treatment for cytopenia, and along with the CD4^+^ T-cell count, the blood cell count was also increased. Persistent cytopenia after 24 months of HAART was associated with femaleness, coinfection with HBV, a CD4^+^ T-cell count at baseline, and WHO stage IV at baseline. Early initiation of HAART and combination antiretroviral therapy without AZT seems to promote the recovery of HIV-infected patients with cytopenia.

## Figures and Tables

**Table 1:** The demographic and clinical characteristics of participants.

Characteristics	Total (*n* = 4325)	Noncytopenia (*n* = 3501)	Cytopenia (*n* = 824)
Age (years)			
<40	2796 (64.6%)	2442 (67.6%)	354 (49.8%)
≥40	1530 (35.4%)	1173 (32.4%)	357 (50.2%)
Sex			
Male	4048 (93.6%)	3340 (95.4%)	708 (85.9%)
Female	277 (6.4%)	161 (4.6%)	116 (14.1%)
WHO stage			
I	3307 (76.5%)	2795 (79.8%)	512 (62.1%)
II	234 (5.4%)	188 (5.4%)	46 (5.6%)
III	281 (6.5%)	217 (6.2%)	64 (7.8%)
IV	503 (11.6%)	301 (8.6%)	202 (24.5%)
BMI (kg/m^2^)			
<18.5	337 (9.8%)	240 (8.1%)	97 (19.7%)
18.5–24	2248 (65.3%)	1938 (65.7%)	310 (62.9%)
>24	858 (24.9%)	772 (26.2%)	86 (17.4%)
CD4^+^ T-cell counts (/*μ*l)			
<200	1475 (35.6%)	949 (22.9%)	526 (66.2%)
≥200	2667 (64.4%)	2398 (71.6%)	269 (33.8%)
Viral load (copies/ml)			
≥100000	979 (30%)	699 (26.2%)	280 (48%)
<100000	2280 (70%)	1977 (73.9%)	303 (52%)
Infection routes			
Sexual	3933 (91%)	3258 (93.1%)	675 (81.9%)
Others	392 (9.9%)	243 (6.9%)	149 (18.1%)
HCV-coinfection			
No	3481 (80.5%)	2934 (81.2%)	547 (76.9%)
Yes	77 (19.5%)	681 (15.7%)	164 (23.1%)
HBV-coinfection			
No	4111 (95%)	3474 (96.1%)	637 (89.6%)
Yes	215 (5%)	141 (3.9%)	74 (10.4%)

Note: data are presented as *n* (%); WHO, World Health Organization; BMI, body mass index; variable had missing values: BMI = 883; HIV RNA = 1066; CD4^+^ T-cell counts = 183.

**Table 2:** The risk factors of cytopenia in HAART-naïve patients.

	df	Univariate analysis OR (95% CI)	*p* value	Multivariate analysis AOR (95% CI)	*p* value
Age (years)	1				
<40		Ref	—	Ref	—
≥40		1.5 (1.21, 1.9)	<0.01	1.51 (1.21, 1.9)	<0.01
Sex	1				
Male		Ref	—	Ref	—
Female		2.9 (2, 4.3)	<0.001	2.92 (2, 4.28)	<0.001
WHO stage	3		<0.001		<0.001
I		Ref	—	Ref	—
II		0.73 (0.44, 1.22)	0.064	0.73 (0.44, 1.22)	0.23
III		1.03 (0.67, 1.59)	0.65	1.03 (0.67, 1.59)	0.9
IV		1.94 (1.45, 2.58)	<0.001	1.94 (1.45, 2.57)	<0.001
HCV-coinfection	1				
No		Ref	—	Ref	—
Yes		1.8 (1.18, 2.76)	0.015	1.8 (1.12, 2.87)	0.007
CD4 (cells/*μ*l)	1				
≥200		Ref	—	Ref	—
<200		3.57 (2.8, 4.5)	<0.001	3.57 (2.83, 4.5)	<0.001
HIV RNA	1				
<100000		Ref	—	Ref	—
≥100000		1.55 (1.24, 2.58)	<0.001	1.55 (1.24, 1.92)	<0.001
BMI	2		0.034		0.034
18.5–24		Ref	—	Ref	—
<18.5		1.6 (1.12, 2.31)	0.01	1.61 (1.1, 2.32)	0.01
>24		1.2 (0.9, 1.54)	0.23	1.18 (0.9, 1.54)	0.23
Infection routes	1				
Others		Ref	—		
Sexual		1.27 (0.6, 2.65)	0.53		
HCV-coinfection	1				
No		Ref	—		
Yes		1.94 (0.98, 3.82)	0.056		

Note: df, degree of freedom; WHO, World Health Organization; BMI, body mass index; Ref, reference.

**Table 3:** The ordinal logistic regression model of cytopenia in HAART-naïve patients.

	B	AOR (95% CI)	*p* value
Age (years)			
<40	Ref	—	—
≥40	0.44	1.55 (1.25, 1.93)	<0.001
Sex			
Male	Ref	—	—
Female	1.064	2.9 (2, 4.3)	<0.001
WHO stage			
I	Ref	—	—
II	−0.3	0.74 (0.45, 1.23)	0.249
III	0.071	1.07 (0.7, 1.65)	0.74
IV	0.748	2.11 (1.6, 2.79)	<0.001
HBV-coinfection			
No	Ref	—	—
Yes	0.58	1.8 (1.18, 2.71)	0.01
CD4 (cells/*μ*l)			
≥200	Ref	—	—
<200	1.29	3.64 (2.9, 4.58)	<0.001
HIV RNA			
<100000	Ref	—	—
≥100000	0.47	1.6 (1.28, 2)	<0.001
BMI			
18.5–24	Ref	—	—
<18.5	0.3	1.4 (1, 1.82)	0.048
>24	−0.145	0.9 (0.66, 1.13)	0.28
Infection routes			
Others	Ref	—	—
Sexual	−0.246	0.78 (0.6, 1.11)	0.17
HCV-coinfection			
No	Ref	—	—
Yes	0.51	1.66 (0.85, 3.22)	0.14

Note: WHO, World Health Organization; BMI, body mass index; Ref, reference.

**Table 4:** The proportion of cytopenias at baseline and 6, 12, and 24 months after HAART.

Cytopenia	Baseline	At 6 months of HAART	At 12 months of HAART	At 24 months of HAART
Neutropenia	285 (46.6%)	97 (18%)	92 (15.1%)	69 (11.3%)
Anemia	349 (57.1%)	91 (14.9%)	56 (9.2%)	48 (7.9.%)
Thrombocytopenia	118 (19.3%)	27 (4.4%)	26 (4.3%)	15 (2.5%)
Unicytopenia	483 (79.1%)	155 (25.4%)	133 (21.8%)	108 (17.7%)
Bicytopenia	111 (18.2%)	21 (3.4%)	16 (2.6%)	12 (2%)
Pancytopenia	16 (2.6%)	6 (1%)	3 (0.5%)	1 (0.2%)

**Table 5:** The risk factors of cytopenia with persistent cytopenia after 24 months HAART.

	df	Univariate analysis, OR (95% CI)	*p* value	Multivariate analysis, OR (95% CI)	*p* value
AZT	1				
No		Ref	—	Ref	—
Yes		4.89 (1.49, 16.07)	0.009	3.71 (1.16, 11.9)	0.009
Sex	1				
Male		Ref	—	Ref	—
Female		5.42 (2.35, 12.5)	<0.001	5.54 (2.47, 12.42)	<0.001
HBV-coinfection	1				
No		Ref	—	Ref	—
Yes		3.8 (1.05, 13.95)	0.042	4.84 (1.47, 15.91)	0.01
CD4^+^T-cell counts at baseline	1				
≥200		Ref	—	Ref	—
<200		2.47 (1.04, 5.85)	0.04	2.42 (1.2, 4.86)	0.013
CD4^+^ T-cell counts at 24 months of HAART	1				
≥200		Ref	—		
<200		0.58 (0.23, 1.48)	0.257		
WHO stage at baseline	3		0.4		
I		Ref	—		
II		1.13 (0.51, 2.5)	0.77		
III		4.37 (0.93, 20.54)	0.124		
IV		0.72 (0.12, 4.21)	0.72		
Age	1				
<40		Ref	—		
≥40		0.97 (0.44, 2.15)	0.93		
D4T	1				
No		Ref	—		
Yes		1.4 (0.72, 2.73)	0.32		
HCV-coinfection	1				
No		Ref	—		
Yes		1.01 (0.1, 9.96)	0.993		

Note: df, degree of freedom; WHO, World Health Organization; BMI, body mass index; Ref, reference; D4T, stavudine; AZT, zidovudine.

## Data Availability

The clinical data used to support the findings of this study have not been made available because of patient privacy.
